# Characterisation and expression analysis of cathepsins and ubiquitin-proteasome genes in gilthead sea bream (*Sparus aurata*) skeletal muscle

**DOI:** 10.1186/s13104-015-1121-0

**Published:** 2015-04-15

**Authors:** Cristina Salmerón, Isabel Navarro, Ian A Johnston, Joaquim Gutiérrez, Encarnación Capilla

**Affiliations:** Department of Physiology and Immunology, Faculty of Biology, University of Barcelona, Av. Diagonal 643, Barcelona, 08028 Spain; Scottish Oceans Institute, School of Biology, University of St Andrews, St Andrews Fife, KY16 8LB Scotland UK

**Keywords:** White muscle, Ontogeny, Life-history stages, Proteases, Lysosomal proteolysis, Ubiquitin-proteasome pathway, Fasting/re-feeding, Teleosts

## Abstract

**Background:**

The proteolytic enzymes involved in normal protein turnover in fish muscle are also responsible for *post-mortem* softening of the flesh and are therefore potential determinants of product quality. The main enzyme systems involved are calpains, cathepsins, and the ubiquitin-proteasome (UbP). In this study on *Sparus aurata* (Sa), the coding sequences of cathepsins (SaCTSB and SaCTSDb) and UbP family members (SaN3 and SaUb) were cloned from fast skeletal muscle, and their expression patterns were examined during ontogeny and in a fasting/re-feeding experiment.

**Results:**

The amino acid sequences identified shared 66-100% overall identity with their orthologues in other vertebrates, with well conserved characteristic functional domains and catalytic residues. SaCTSDb showed phylogenetic, sequence and tissue distribution differences with respect to its paralogue SaCTSDa, previously identified in the ovary. Expression of gilthead sea bream cathepsins (B, L, Da, Db) and UbP members (N3, Ub, MuRF1 and MAFbx) in fast skeletal muscle was determined at three different life-history stages and in response to fasting and re-feeding in juveniles. Most of the proteolytic genes analysed were significantly up-regulated during fasting, and down-regulated with re-feeding and, between the fingerling (15 g) and juvenile/adult stages (~50/500 g), consistent with a decrease in muscle proteolysis in both later contexts. In contrast, SaCTSDa and SaMuRF1 expression was relatively stable with ontogeny and SaUb had higher expression in fingerlings and adults than juveniles.

**Conclusions:**

The data obtained in the present study suggest that cathepsins and UbP genes in gilthead sea bream are co-ordinately regulated during ontogeny to control muscle growth, and indicate that feeding regimes can modulate their expression, providing a potential dietary method of influencing *post-mortem* fillet tenderisation, and hence, product quality.

**Electronic supplementary material:**

The online version of this article (doi:10.1186/s13104-015-1121-0) contains supplementary material, which is available to authorized users.

## Background

Gilthead sea bream (*Sparus aurata* L.) is widely farmed around the Mediterranean area [1] and because of its commercial importance, muscle growth regulation in this species has been the subject of recent research [2-9]. In common with most teleosts, gilthead sea bream exhibits indeterminate growth, with muscle mass increasing by hyperplasia (production of new fibres) until 40-50% of the maximum length, and by hypertrophy (increase of fibres size) until mortality or senescence occur [10,11].

Muscle growth represents the balance between protein synthesis and degradation, and sarcomeric components have a range of half-lives [12]. In vertebrates, four catabolic systems are known to be involved in muscle proteolysis: a) the Ca^2+^-dependent proteinases (calpains), b) the autophagy-lysosome system (cathepsins), c) the ATP-dependent ubiquitin-proteasome (UbP) pathway and, d) the apoptosis protease system (caspases) [13-16]. Calpains are considered a system of primary protein degradation, with a regulatory or signalling function, since they do not cleave proteins to amino acids or small peptides [17]. Cathepsins and the UbP pathway are required for the complete degradation of protein substrates [18].

The UbP pathway operates through a multi-subunit proteolytic complex, the proteasome, and it targets specific proteins for destruction through a three-step enzymatic process that covalently links a poly-ubiquitin (Ub) chain to the protein substrate to be degraded. Two E3 Ub ligases, MuRF1 and MAFbx (also known as Atrogin-1), are considered transcriptional markers involved in muscle wasting in vertebrates; however, they seem to contribute differently to muscle loss. MuRF1 is involved in the breakdown of myofibrillar proteins such as myosin heavy chain, whereas MAFbx participates in the control of protein synthesis, regulating transcription factors such as MyoD [19,20]. The UbP pathway in vertebrates is particularly important during muscle atrophy, either caused by starvation or wasting diseases [21-25], and it is also involved in the age-related loss of muscle mass (sarcopenia) in mammals [26,27]. In fish, prolonged fasting has been shown to induce muscle atrophy [28-30], whereas signs of sarcopenia as the fish age have been reported only for species with determinate growth such as the zebrafish (*Danio rerio*) [31]. Previous studies in rainbow trout (*Oncorhynchus mykiss*) have shown that fasting increased 20S proteasome activity [32] whereas the mRNA expression of several UbP pathway members decreased with re-feeding [33,34]. In the same species, UbP gene expression was unchanged following muscle wasting associated with spawning [16,35].

Cathepsins are lysosomal proteases optimally active in a slightly acidic environment, and are classified as aspartic, serine or cysteine proteases according to the amino acid found in their active site [36]. The role of cathepsins in muscle proteolysis and *post-mortem* degradation has been investigated in several fish species. Calpains and cathepsins are thought to be involved in enzymatic degradation of key structural and extracellular matrix proteins during *post-mortem* tenderisation [37]. In sockeye salmon (*Oncorhynchus nerka*), the muscle protein degradation that occurs during the final stages of maturation following migration and fasting involves lysosomal cathepsin D, and to a lesser extent, cathepsin L [38]. In rainbow trout, fasting and re-feeding respectively increased and decreased the mRNA expression of cathepsins B, D and S [33], and spawning-induced muscle wasting was associated with increased cathepsin L and D mRNA levels [16,35]. In gilthead sea bream, distinct changes in the expression and enzyme activities of cathepsins B, D and L have been observed in the maturing ovary, indicating a specific function for these enzymes during the follicle maturation process [39-41].

Following slaughter the endogenous activity of proteolytic enzymes contributes to *post-mortem* softening and a loss of product quality [37,42,43]. We have recently characterised several members of the calpain system in gilthead sea bream muscle, and demonstrated that their expression is modulated by nutritional status and diet composition [44]. In the present study, complete and partial coding sequences (CDS) for the cathepsins (SaCTSB and SaCTSDb) and UbP family members (SaN3 and SaUb) were cloned from fast skeletal muscle, and their expression patterns examined during ontogeny and with fasting and re-feeding.

## Methods

### Ethical statement

All animal handling procedures were approved by the Ethics and Animal Care Committee of the University of Barcelona (CEEA 239/09) and the “Departament de Medi Ambient i Habitatge” (DMAH permit number 5420, Generalitat de Catalunya, Spain) following the European Union, Spanish and Catalan Government established norms and procedures.

### Fish and experimental trials

Fish used for the cloning and expression analysis during ontogeny were obtained from a fish farm in Northern Spain. Gilthead sea bream for the fasting/re-feeding experiment were obtained from the “Institut de Recerca i Tecnologia Agroalimentàries” (IRTA) facilities (Sant Carles de la Ràpita, Spain). All fish were acclimatized to the facilities at the University of Barcelona (Barcelona, Spain) for a minimum of two weeks prior to sampling or experimental manipulations, fed *ad libitum* twice daily with commercial pellets (Excel, Skretting, Burgos, Spain) and held at 21 ± 1°C (range) and pH 7.5-8 in 200 or 400 L recirculating seawater tanks with 12 h dark: 12 h light photoperiod. For cloning 10 juvenile gilthead sea bream of 43 ± 3 g and for the tissue screening 6 juveniles of 151 ± 12 g (mean ± SEM) were used. For the life-history stages study, groups of 5 fish each of 15 ± 1 g (fingerlings, FL), 47 ± 5 g (juveniles, JV) and 503 ± 37 g (adults, AD) (mean ± SEM) were used. For the fasting/re-feeding experiment 120 juvenile gilthead sea bream (50 ± 6 g) were used, and the trial performed as previously described [44] (mean ± SEM). First, acclimatized fish were sampled for time 0 (D0C) and then, divided into two conditions: control fed group at 3% (m/m d^−1^) body weight (C) and a fasted group (F). Samples were collected at days 15 and 30 (D15C/F and D30C/F) and subsequently, the remaining fasted animals (D0R) were re-fed at 2% (m/m d^−1^) body weight (lower ration than the control to facilitate correct adaptation of the digestive system) and sampled at days 7 and 14 (D7R and D14R). Before sampling all fish were fasted 24 h, anesthetized with tricaine methane sulphonate (MS-222 0.1 g/L, Sigma, Tres Cantos, Spain) and sacrificed with a blow on the head and medullar section. Samples of fast skeletal muscle (FM), slow skeletal muscle (SM), heart (HE), brain (BR), liver (LI), spleen (SP) and immature gonad (GO) were taken by sterile dissection and immediately snap-frozen in liquid nitrogen and stored at −80°C for cloning or gene expression analyses.

### RNA extraction and cDNA synthesis

Total RNA was extracted from 20–130 mg of tissue following the guanidinium thiocyanate-phenol-chloroform method [45] using TRIreagent (Applied Biosystems, Alcobendas, Spain), quantified using a NanoDrop2000 spectrophotometer (Thermo Scientific, Alcobendas, Spain) and, RNA quality was analysed by 1% (m/v) agarose gel electrophoresis. One μg of total RNA per sample was used to synthesise first-strand cDNA using the Transcriptor First Strand cDNA Synthesis Kit (Roche, Sant Cugat del Vallès, Spain) or the AffinityScript QPCR cDNA Synthesis Kit (Agilent Technologies, Las Rozas, Spain) in the case of the tissue screening, following the manufacturers’ instructions. cDNA samples were diluted 1:5 in milliQ H_2_O for conventional polymerase chain reaction (PCR) and diluted 1:40 to 1:100 in milliQ H_2_O for real-time quantitative PCR (qPCR).

### Cloning and sequencing

To obtain the complete sequences of gilthead sea bream (Sa) cathepsin B (SaCTSB), the new paralogue of cathepsin D (SaCTSDb), the proteasome beta type-4 subunit (SaN3, also known as PSMB4) and the ubiquitin (SaUb) from fast skeletal muscle, specific primers for PCR were designed using the DNAman software package (Lynnon, Quebec, Canada) and Net primer (Premier BioSoft) (www.premierbiosoft.com/netprimer/) using the gilthead sea bream ESTs (Expressed Sequence Tags) from the NCBI database (SaCTSB: [GenBank: HS985610] and [GenBank: FG26781]; SaCTSDb: [GenBank: FM146030] and [GenBank: FG26194]; SaN3: [GenBank: HS988518]; SaUb: [GenBank: AM955423]) as templates (Table 1). The cloning was performed as previously described [44]. Briefly, PCR products were separated by gel electrophoresis and purified from the agarose gel using the PureLink Quick Gel Extraction Kit (Invitrogen, Alcobendas, Spain). The purified PCR product was ligated into T/A pCR4-TOPO vector and transformed into chemically competent TOP10 *Escherichia coli* cells (all from Invitrogen, Alcobendas, Spain). One to three clones of each PCR product were sequenced using BigDye Terminator v3.1 Cycle Sequencing Kit (Applied Biosystems, Alcobendas, Spain) and analysed at the “Serveis Cientificotècnics” of the University of Barcelona (Barcelona, Spain). Sequenced products were joined *in silico* using the sequence alignment editor and sequence analysis program BioEdit [46] to produce contigs with a single open reading frame (ORF). Sequences generated were analysed for similarity with other known sequences using the BLAST programs (http://blast.ncbi.nlm.nih.gov/Blast.cgi).

**Table 1:** **Primer sequences for cloning**

**Primers ID**	**Primer sequences (5’-3’)**	**GenBank**	**Ta (°C)**	**Amplicon (bp)**
SaCTSDb_FW	TCGGACTGTTACGATGAGGA	FM146030	56	1240
SaCTSDb_RV	CTTTGCACTTGGACGAGTTG	FG26194		
SaCTSB_FW	CCCGAAGATTATAACCAAGTTGAC	HS985610	59	1249
SaCTSB_RV	GTGACTTGTGCTCAGAAACGTAGT	FG26781		
SaUb_FW	CGGAAGTAAGAGGAACCAACAC	AM955423	56	1132
SaUb_RV	AAGCAGTCAGAATGCAAAGTCA			
SaN3_FW	CAGGTTTGAAGCTGAGTTTCTG	HS988518	58	759
SaN3_RV	CTGACCATGTGAGCGATGTC			

Primer sequences used to clone the cathepsins (SaCTSDb and SaCTSB) and ubiquitin-proteasome members (SaUb and SaN3) from gilthead sea bream (Sa) fast muscle. FW: forward, RV: reverse; Ta: annealing temperature; bp: base pair.

### Sequence and phylogenetic analyses

The putative protein architecture (domains, active sites and other important motifs) from the sequences generated was determined according to the literature and the conserved domain search program of NCBI [47] and the simple modular architecture research tool (SMART) version 4.0 (http://smart.embl-heidelberg.de) [48]. Compute pI/Mw tool (ExPASy, Switzerland, http://www.expasy.org/tools/pi_tool.html) was used to estimate the molecular weight (Mw) of the predicted proteins and NetNGlyc 1.0 Server (http://www.cbs.dtu.dk/services/NetNGlyc/) was used to predict the N-Glycosylation sites. All the alignments were created with MAFFT version 7.058b (http://mafft.cbrc.jp/alignment/software/) and G-INS-i (recommended for <200 sequences with global homology) strategy. Sequences used in the present study other than those cloned from gilthead sea bream fast skeletal muscle were obtained from NCBI. Phylogenetic analyses, including multiple sequence alignment and Maximum Parsimony (MP) tree prediction of 27 vertebrate cathepsin orthologs of CTSB, CTSD and CTSL were conducted using MEGA version 5.0 [49]. MP trees were obtained using the Close-Neighbour-Interchange algorithm and bootstrap values were inferred from 1000 replicates. The gilthead sea bream calpain 1 [GenBank: KF444899], a cytosolic cysteine protease, was used to root the phylogenetic tree.

### Conventional PCR

The mRNA levels of both SaCTSD paralogues under normal physiological conditions were measured in gilthead sea bream tissues using qualitative PCR and elongation factor 1-α (SaEF1α) was used as a loading control. Reactions were performed in a final volume of 50 μL, containing 1 μL of first-strand cDNA (equivalent to 4 ng of reverse transcribed total RNA), 1.5 U of Taq polymerase (Sigma, Tres Cantos, Spain) and 200 nM (final concentration) of sense and antisense primers (Table 2). Reactions proceeded in a MyiQ Thermal Cycler (Bio-Rad, El Prat de Llobregat, Spain) with the following protocol: 1 cycle at 95°C for 5 min, 35 cycles at 95°C for 30 s, 56-60°C (primer dependent, see Table 2) for 30 s, 72°C for 30 s and 1 cycle at 72°C for 7 min. Each reaction product was separated by agarose gel electrophoresis and visualised using SYBR Safe DNA gel stain (Life Technologies, Alcobendas, Spain) in a LAS-3000 (Fujifilm, Madrid, Spain) to confirm that a single product was amplified, and then sequenced to confirm the specificity of each assay as explained above in 2.4. Semi-quantification of SaCTSD paralogues relative expression normalized to SaEF1α was performed determining band intensity using the ImageJ software version 1.47 (National Institutes of Health, Bethesda, USA).

**Table 2:** **Primer sequences for qualitative and quantitative PCR**

**Primers ID**	**Primer sequences (5’-3’)**	**GenBank**	**Ta (°C)**	**Amplicon (bp)**
SaCTSDa_qFW	CCTCCATTCACTGCTCCTTC	AF036319	56	107
SaCTSDa_qRV	ACCGGATGGAAAACTCTGTG			
SaCTSDb_qFW	AAATTCCGTTCCATCAGACG	KJ524456	56	131
SaCTSDb_qRV	CTTCAGGGTTTCTGGAGTGG			
SaCTSL_qFW	ACTCCTTGGGCAAACACA	DQ875329	54	116
SaCTSL_qRV	CCTTGAACTTCCTCTCCGT			
SaCTSB_qFW	GCAGCCTTCCTGTTATTGG	KJ524457	57	185
SaCTSB_qRV	AGGTCCCTTCAGCATCGTA			
SaUb_qFW	ACTGGCAAGACCATTACCTT	KJ524459	54	160
SaUb_qRV	TGGATGTTGTAGTCGGAAAG			
SaN3_qFW	AGACACACACTGAACCCGA	KJ524458	54	118
SaN3_qRV	TTCCTGAAGCGAACCAGA			
SaMuRF1_qFW	GTGACGGCGAGGATGTGC	FM145056	60	50
SaMuRF1_qRV	CTTCGGCTCCTTGGTGTCTT			
SaMAFbx_qFW [2]	GGTCACCTGGAGTGGAAGAA	ERA047531	60	158
SaMAFbx_qRV [2]	GGTGCAACTTTCTGGGTTGT			
Saβ-actin_qFW [44]	TCCTGCGGAATCCATGAGA	X89920	60	50
Saβ-actin_qRV [44]	GACGTCGCACTTCATGATGCT			
Sa18S_qFW(a) [82]	CTCAACACGGGAAACCTCACC	NR_003286	56	119
Sa18S_qRV(a) [82]	CAGACAAATCGCTCCACCAACTA			
Sa18S_qFW(b) [83]	TGACGGAAGGGCACCACCAG	AY550956	60	158
Sa18S_qRV(b) [83]	AATCGCTCCACCAACTAAGAACGG			
SaEF1α_qFW [44]	CTTCAACGCTCAGGTCATCAT	AF184170	60	263
SaEF1α_qRV [44]	GCACAGCGAAACGACCAAGGGGA			

Primer sequences for gilthead sea bream (Sa) cathepsins (SaCTSDa, SaCTSDb, SaCTSL and SaCTSB), ubiquitin-proteasome members (SaUb, SaN3, SaMuRF1 and SaMAFbx) and reference genes (Saβ-actin, Sa18S and SaEF1α) used for conventional PCR and qPCR analyses. qFW: forward, qRV: reverse; Ta: annealing temperature; bp: base pair. (a) Sa18S primers used in the life-history stages and (b) the tissue screening experiments.

### Quantitative real-time PCR (qPCR)

To characterise the two cathepsin D paralogues in gilthead sea bream, mRNA abundance in fast skeletal muscle and immature gonad from the tissue screening samples was evaluated using qPCR. mRNA levels of the different cathepsins and UbP genes in fast skeletal muscle from different life-history stages and the fasting/re-feeding experiment were also determined. In addition, Saβ-actin, the 18S ribosomal RNA (Sa18S) and SaEF1α were tested as reference genes. The specific gilthead sea bream qPCR primers not previously reported (see Table 2) were designed using the DNAman software package and Net primer (Premier BioSoft) (www.premierbiosoft.com/netprimer/). The qPCR assay was conducted as previously described [44]. Briefly, reactions contained first-strand cDNA (equivalent to 2.5 ng of reverse transcribed total RNA), iQ SYBR Green Supermix (Bio-Rad, El Prat de Llobregat, Spain) and 250 nM (final concentration) of sense and antisense primers (Table 2), and were performed in triplicate using a MyiQ or a CFX384 thermocycler (Bio-Rad, El Prat de Llobregat, Spain). The protocol consisted on 1 cycle of 3 min at 95°C and 40 cycles of 10 s at 95°C and 30 s at 54-60°C (primer dependent, see Table 2), followed by an amplicon dissociation analysis from 55 to 95°C at 0.5°C increase each 30 s, where a single peak was observed confirming the specificity of the reaction. SYBR Green fluorescence was recorded during the annealing-extending phase of cycling. Expression results were normalized to Saβ-actin, the most stable of the three reference genes tested, and analysed by the delta-delta method [50].

### Statistical analyses

Statistical analyses of all parameters were performed in PASW Statistics 17.0 (IBM, Chicago, USA). Normality was analysed according to the Shapiro-Wilk test and homogeneity in variance according to Levene’s test. Statistical differences among groups in the ontogeny as well as the fasting and re-feeding experiments were assessed by one-way ANOVA, followed by Tukey’s *post hoc* test, or between the two SaCTSD paralogues in the tissue expression analysis by Student’s t-test. A significance of p < 0.05 was applied to all statistical tests performed. Data are presented as mean ± standard error of the mean (SEM).

## Results

### Sequence analysis of SaCTSB, SaCTSDb, SaN3 and SaUb

**Figure 1:**
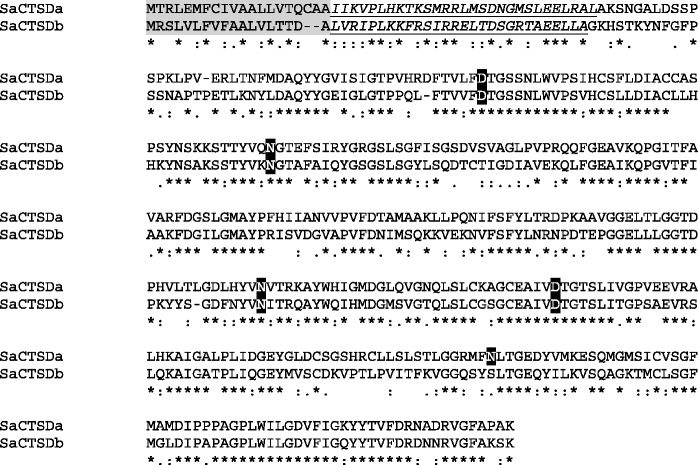
Sequence comparison of gilthead sea bream cathepsin D paralogues. Alignment of deduced amino acid sequence of both gilthead sea bream (Sa) cathepsin D paralogues; SaCTSDa [GenBank: AF036319] and SaCTSDb [GenBank: KJ524456], via MAFFT (v7.058b) and G-INS-I method. Symbols: (*) identical residues in both sequences; (:) conservative substitutions and (.) semiconservative substitutions. The putative signal peptide sequence is highlighted in grey and the propeptide region (A1_Propeptide) is underlined and in italics. The predicted N-glycosylation sites (N) and the two aspartyl (D) conserved catalytic residues are shaded in black.

The CDS of the first cathepsin identified, SaCTSB, consisted of 993 base-pairs (bp) that encode a protein of 330 amino acids (aa) with a putative molecular mass (Mw) of 36.41 kDa [GenBank: KJ524457]. BLAST analysis showed that SaCTSB shares 70-82% overall sequence identity with the cathepsin B proteins of a number of fish, amphibian and mammalian species (Table 3). *In silico* analysis identified in SaCTSB an N-terminal signal peptide (I29/Propeptide C1), a cathepsin B propeptide region, and a papain family cysteine protease domain (Additional file 1: Figure S1). The protease domain contains the four essential residues for catalysis, i.e. Q101, C107, H277 and N297, which are highly conserved among vertebrates plus a predicted N-glycosylation site located at position 190. The CDS of the second cathepsin cloned, SaCTSDb, was 1191 bp encoding a 396 aa protein with a putative Mw of 42.98 kDa [GenBank: KJ524456]. SaCTSDb consists of a putative N-terminal signal peptide, a cathepsin D propeptide region (A1_Propeptide), and an aspartyl protease domain (Additional file 1: Figure S2), with the two aspartyl, D94 and D281 catalytic residues, conserved. A comparison of the SaCTSDb with other vertebrate cathepsin D protein sequences revealed a high degree of sequence similarity (66-88%; Table 3). Moreover, the sequence analysis of SaCTSDb predicted two possible N-glycosylation sites, located at positions 131 and 249 (Figure 1). Compared to the previously reported cathepsin D in gilthead sea bream ovary (SaCTSDa), the newly identified SaCTSDb has 57% aa identity, but lacks the third residue of N-glycosylation at position 337 (Figure 1). The partial CDS of SaN3 (97% of the putative molecule) was 756 bp and encoded 252 aa [GenBank: KJ524458]. SaN3 showed high levels of identity (74-93%) with other vertebrate proteasome N3/PSMB4 sequences (Table 3). The partial SaN3 protein sequenced contains the proteasome domain with threonine endopeptidase activity and shows the characteristic aa in the active site and interaction site typical of proteasome beta type-4 subunits (Additional file 1: Figure S3). Finally, we identified SaUb with a CDS of 918 bp, encoding a 305 aa protein with a putative Mw of 34.32 kDa [GenBank: KJ524459]. Four identical aa repeat units, termed R1 to R4, were present in the SaUb sequence each consisting of 216 bp coding for a 72 aa Ub monomer (Additional file 1: Figure S4). Each Ub monomer showed an interaction site with the Ub-conjugating enzyme (E2) and also an interaction site with the C-terminal hydrolase (UCH), as well as binding sites to the CUE domain of the Cue2 protein (Additional file 1: Figure S4). The protein sequence of SaUb was 100% identical to all vertebrate Ub protein sequences analysed (Table 3).

**Table 3:** **Comparison of amino acid sequences identities**

	***M. musculus***	***X. laevis***	***T. rubripes***	***D. rerio***	***S. salar***
SaCTSB	71	72	70	81	82
SaCTSL	63	76	88	83	84
SaCTSDa	58	58	58	66	73
SaCTSDb	66	77	88	83	87
SaN3	74	80	93	90	90
SaUb	100	100	100	100	100

Percentages of amino acid sequence identity between the gilthead sea bream (Sa) and the *Mus musculus*, *Xenopus laevis*, *Takifugu rubripes*, *Danio rerio* and *Salmo salar* cathepsins (B, L, Da and Db) and the ubiquitin-proteasome members (N3 and Ub).

### Phylogenetic analysis of SaCTSs

**Figure 2:**
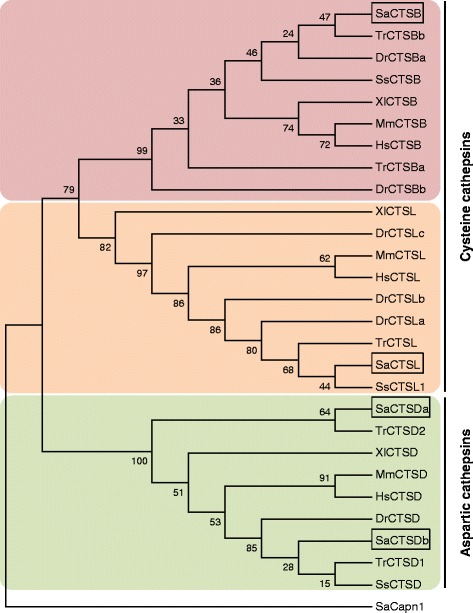
Phylogenetic analysis of gilthead sea bream cathepsins B, D and L. Rooted Maximum Parsimony tree predicting the evolutionary relationship between *Sparus aurata* (Sa), *Danio rerio* (Dr), *Homo sapiens* (Hs), *Mus musculus* (Mm), *Salmo salar* (Ss), *Takifugu rubripes* (Tr) and *Xenopus laevis* (XI) CTSB, CTSD and CTSL orthologues. The calpain 1 sequence of gilthead sea bream (SaCapn1) [GenBank: KF444899] was used as outgroup. Bootstrap values, calculated from 1000 replicates, are indicated at the nodes. Gilthead sea bream cathepsins are boxed. The NCBI GenBank accession numbers for the analysed sequences are: *D. rerio* (DrCTSBa: NM_213336; DrCTSBb: NM_001110478; DrCTSD: NM_131710; DrCTSLa: BC066490; DrCTSLb: NM_131198), *H. sapiens* (HsCTSB: L16510; HsCTSD: NM_001909; HsCTSL: NM_001912), *M. musculus* (MmCTSB: NM_007798; MmCTSD: NM_009983; MmCTSL: NM_009984), *S. salar* (SsCTSB; NM_001140522; SsCTSD: BT043515; SsCTSL1: NM_001146546), *S. aurata* (SaCTSB: KJ524457; SaCTSDa: AF036319; SaCTSDb: KJ524456 and; SaCTSL: DQ875329), *T. rubripes* (TrCTSBa: XM_003971718; TrCTSBb: XM_003969499; TrCTSD1: AB179548; TrCTSD2: AB179549; TrCTSL: XM_003975074) and *X. laevis* (XlCTSB: NM_001086101; XlCTSD: AB103479; XlCTSL: NM_001092267).

Phylogenetic analysis of cathepsins B, D and L from representative mammals, amphibians and fish, including SaCTSB, SaCTSDa, SaCTSDb and SaCTSL, produced an MP-phylogenetic tree that contained two distinct branches. The cysteine proteases, cathepsins B and L, clustered together separated from the aspartic proteases, the cathepsin Ds (Figure 2). The SaCTSB, SaCTSL, SaCTSDa and SaCTSDb were clustered together with their orthologous vertebrate cathepsins (Figure 2). The relationships revealed in the phylogenetic tree were in agreement with the concept of traditional taxonomy. In addition, the new cathepsin D paralogue cloned from gilthead sea bream fast skeletal muscle, the SaCTSDb, appeared phylogenetically related to its CTSD orthologues from other teleost and tetrapod species, whereas the other paralogue previously cloned in ovary, SaCTSDa, formed a clade with only CTSD sequences of teleost species (Figure 2).

### Tissue expression of SaCTSDa and SaCTSDb

To further characterise the two cathepsin D paralogues identified, and to see if they have other differences (e.g. tissue distribution) that can help postulate differences in function and explain why they have both been retained throughout evolution, gene expression analyses of a tissue panel was performed.

**Figure 3:**
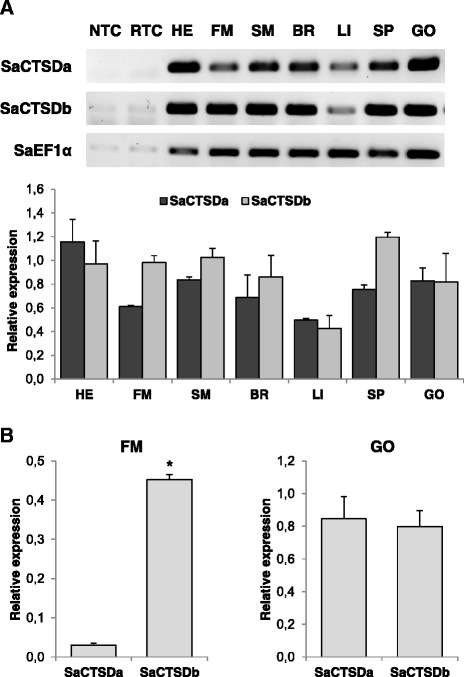
Tissue expression of gilthead sea bream cathepsin D paralogues. **(A)** Qualitative transcript expression profiles of SaCTSDa and SaCTSDb across adult gilthead sea bream (Sa) tissue types: fast skeletal muscle (FM), slow skeletal muscle (SM), heart (HE), brain (BR), liver (LI) spleen (SP) and immature gonad (GO). SaEF1α was also amplified as a reference gene to confirm a steady-state level of expression among tissues. No template (NTC) and no reverse transcriptase (RTC) negative controls were also included to confirm primer specificity and the absence of genomic DNA. Representative image and semi-quantification of band intensity from n = 3 fish. **(B)** Quantitative relative expression normalized to Saβ-actin of SaCTSDa and SaCTSDb from gilthead sea bream (Sa) fast skeletal muscle (left) and gonad (right). Results are shown as mean ± SEM (n = 4-6). The asterisk indicates significant differences at p < 0.05.

Qualitative PCR analysis showed that under normal physiological conditions, both SaCTSDa and SaCTSDb were ubiquitously expressed in all tissues analysed, although SaCTSDb mRNA was relatively more abundant in fast and slow skeletal muscle (Figure 3A). Moreover, both paralogues showed lower levels of expression in the liver compared to the other tissues.

Quantitative analysis of the expression of both paralogues in gilthead sea bream fast skeletal muscle and immature gonad confirmed that SaCTSDb has 15-fold higher expression in muscle than SaCTSDa, whereas a similar level of expression for both paralogues was observed in the gonad (Figure 3B).

### Expression of SaCTSs and SaUbP genes during life-history stages

**Figure 4:**
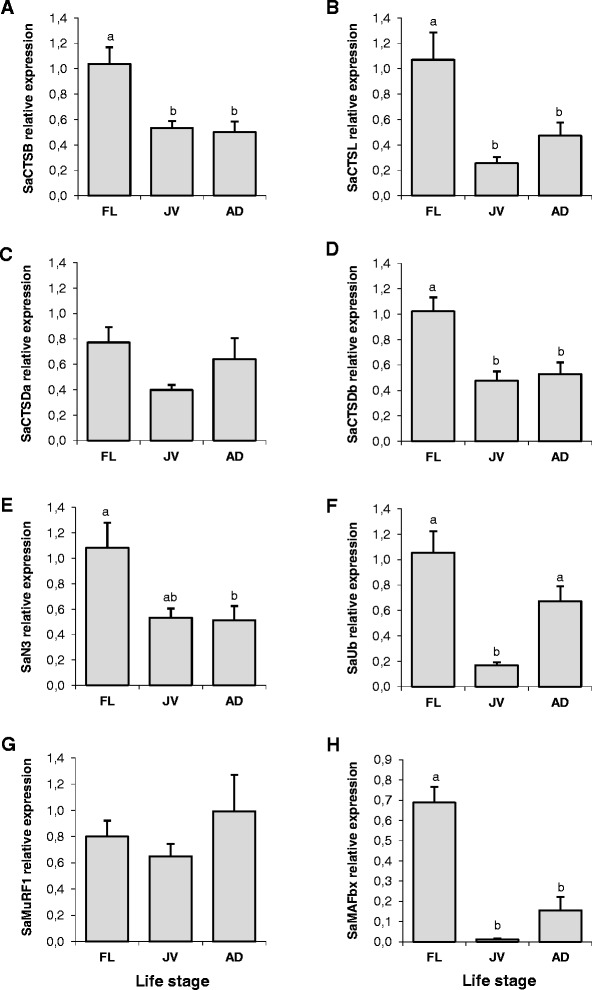
Proteolytic markers expression in gilthead sea bream muscle at different life-history stages. Quantitative relative expression normalized to Saβ-actin of **(A)** SaCTSB, **(B)** SaCTSL, **(C)** SaCTSDa, **(D)** SaCTSDb, **(E)** SaN3, **(F)** SaUb, **(G)** SaMuRF1 and **(H)** SaMAFbx from gilthead sea bream (Sa) fast skeletal muscle from fish at three different life-history stages: fingerlings (FL), juveniles (JV) and adults (AD). Results are shown as mean ± SEM (n = 4-5). Different letters indicate significant differences at p < 0.05.

To study the transcriptional regulation of the different proteolytic members present in the fast skeletal muscle of gilthead sea bream, we first analysed the expression of the SaCTSs and SaUbP members at three different life-history stages. qPCR analysis showed that the mRNA expression of SaCTSB, SaCTSL, SaCTSDb and SaMAFbx decreased significantly as fish grow, being greater in the muscle of fingerlings than in juveniles or adult fish (Figure 4A, B, D and H). SaN3 expression was also down-regulated with ontogeny, being significantly higher in fingerlings than in adult fish (Figure 4E). In contrast, SaUb expression was significantly higher in the muscles of both fingerlings and adult fish relative to juveniles (Figure 4F), whereas the expression of SaCTSDa and SaMuRF1 remained unchanged (Figure 4C and G).

### Expression of SaCTSs and SaUbP genes in response to fasting and re-feeding

**Figure 5:**
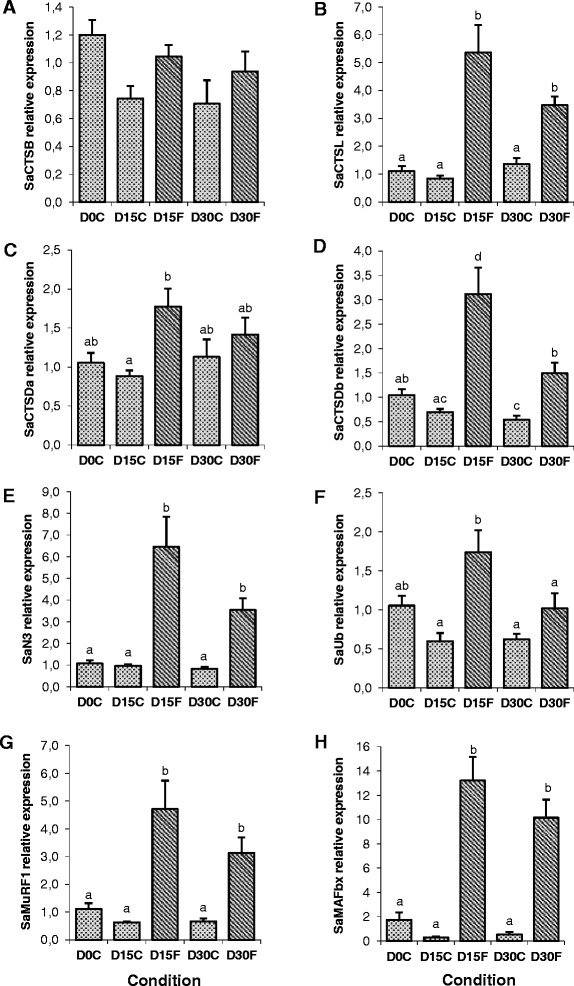
Proteolytic markers expression in gilthead sea bream muscle during fasting. Quantitative relative expression normalized to Saβ-actin of **(A)** SaCTSB, **(B)** SaCTSL, **(C)** SaCTSDa, **(D)** SaCTSDb, **(E)** SaN3, **(F)** SaUb, **(G)** SaMuRF1 and **(H)** SaMAFbx from gilthead sea bream (Sa) fast skeletal muscle from fish at 0, 15 and 30 days of fasting. Results are shown as mean ± SEM (n = 6-8). Different letters indicate significant differences at p < 0.05.

**Figure 6:**
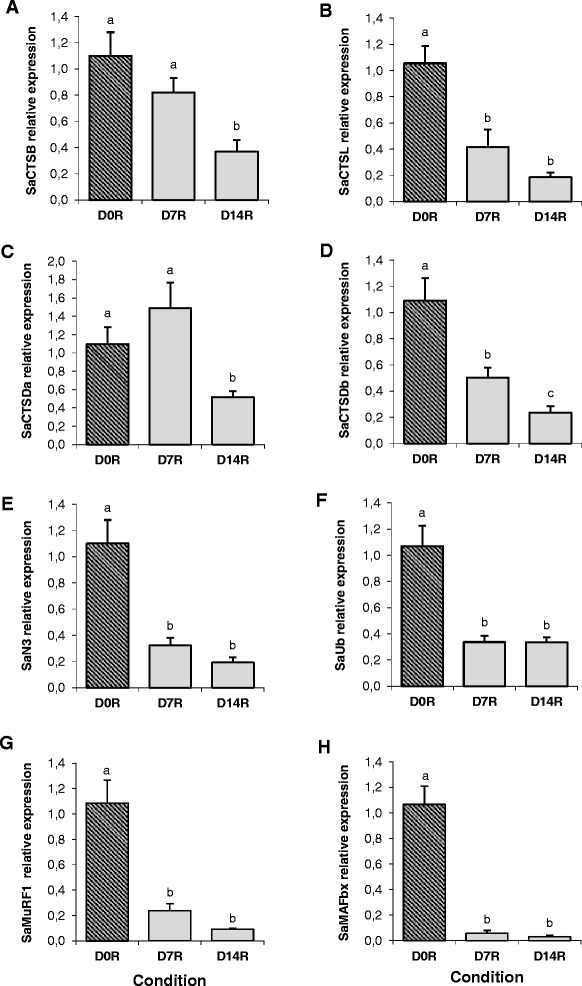
Proteolytic markers expression in gilthead sea bream muscle during re-feeding. Quantitative relative expression normalized to Saβ-actin of **(A)** SaCTSB, **(B)** SaCTSL, **(C)** SaCTSDa, **(D)** SaCTSDb, **(E)** SaN3, **(F)** SaUb, **(G)** SaMuRF1 and **(H)** SaMAFbx from gilthead sea bream (Sa) fast skeletal muscle from fish at 0, 7 and 14 days of re-feeding after a period of 30 days fasting. Results are shown as mean ± SEM (n = 3-8). Different letters indicate significant differences at p < 0.05.

The transcriptional regulation of SaCTSs and SaUbP members was studied in fish skeletal muscle during fasting (15 and 30 days) and subsequent re-feeding for 7 and 14 days. The mRNA levels of all genes analysed were increased during the two time points of fasting except for SaCTSB that remained unchanged (Figure 5A). The gene expression of SaCTSL (Figure 5B), SaCTSDb (Figure 5D), SaN3 (Figure 5E), SaMuRF1 (Figure 5G) and SaMAFbx (Figure 5H) was significantly higher during the whole fasting period compared to the corresponding control condition; whereas SaCTSDa (Figure 5C) and SaUb (Figure 5F) increased at day 15 but decreased again after 30 days of fasting to values similar to those of the control. All the genes studied were down-regulated in response to re-feeding. SaCTSL (Figure 6B), SaCTSDb (Figure 6D), SaN3 (Figure 6E), SaUb (Figure 6F), SaMuRF1 (Figure 6G) and SaMAFbx (Figure 6H) decreased significantly after 7 days of re-feeding and remained low at day 14, while the expression of SaCTSB (Figure 6A) and SaCTSDa (Figure 6C) decreased significantly only after 14 days of re-feeding.

## Discussion

In the present study, the complete CDS of cathepsin B (SaCTSB), a new paralogue of cathepsin D (SaCTSDb), ubiquitin (SaUb), and partially the CDS of the proteasome subunit beta type-4 known as N3 or PSMB4 (SaN3), were cloned from gilthead sea bream fast skeletal muscle. The deduced amino acid sequences obtained, shared high levels of overall identity with orthologues of CTSB, CTSD, Ub and N3/PSMB4 in teleosts and other vertebrate species. For example, SaCTSB and SaCTSDb, possess an N-terminal signal peptide, a propeptide region, and a cysteine or aspartyl catalytic protease domain, respectively, with complete conservation at the catalytic residues [36,51]. SaN3 has a histidine residue replacing the mammalian arginine before the key threonine essential for proteolytic function [52] as previously reported in the N3 sequence of rainbow trout [53]. In order to maintain the subunit in an inactive form until the proteasome is assembled the beta type subunits are synthesized as inactive precursors and activated after autocatalytic internal cleavage [54]. Analysis of the SaN3 sequence also showed that the active site and the beta subunit interaction site were conserved as in other vertebrate N3 sequences [55]. The SaUb cDNA identified had four 228 bp repeats each encoding a 72 amino acids Ub monomer, which has 100% identity with other vertebrate Ub sequences. The final repeat has an additional asparagine, rather than a tyrosine residue as in mammals [56], as has been shown to be the case also in Ub from rainbow trout [53]. The structural features and the high levels of identity with the respective vertebrate sequences indicate that SaN3 and SaUb are the corresponding structural orthologues of N3 and Ub, respectively.

Phylogenetic analysis of cathepsin sequences produced a tree with two main branches; one with the cysteine CTSB and CTSL proteases and the other one with the aspartic CTSDs. The cysteine proteases arose early during evolution and the high percent identity among residues across taxa suggests that both cathepsin subfamilies evolved from gene duplication events [57]. Interestingly, SaCTSDb seems to be the more common and less derived SaCTSD paralogue from the teleost whole genome duplication [58]. Moreover, this new paralogue SaCTSDb clustered with other fish and mammalian cathepsin Ds, whereas SaCTSDa clustered on a separate branch with only fish members.

SaCTSDb lacks the third putative N-glycosylation site present in SaCTSDa whereas other vertebrate sequences possess only one or two, as previously reported [38]. A previous study in human cells with mono-glycosylated and non-glycosylated cathepsin D mutants has shown that glycosylation is not necessary for folding or enzyme activity but it is required for targeting the enzyme to lysosomes [59]. SaCTSDa and b were ubiquitously expressed in all tissues analysed as previously reported in other fish, avian and mammalian species [60-65]. The SaCTSDa gene was expressed in gonad as previously observed, in agreement with its role during the cleavage of vitellogenin to yield yolk proteins [39,62,66-70]. The SaCTSDb gene was more highly expressed in skeletal muscle tissue than SaCTSDa, consistent with it having probably some specific role in the degradation of muscle proteins, which should be addressed in future studies.

In the present study, the expression of most cathepsins and UbP members’ decreased with ontogeny. Dietary protein requirements decrease as the fish grow, e.g. the required protein and energy content in diets are higher in smaller (<100 g) than in larger fish [71]. Therefore, it appears that mRNA levels reflect a decrease in proteolytic activity and protein turnover, where fingerlings have higher proteolysis rates than juvenile and adult fish, as well as higher rates of protein synthesis, as previously shown for example in rainbow trout [72,73]. Interestingly, expression of SaCTSDb and SaMAFbx decreased during ontogeny whereas SaCTSDa and SaMuRF1 expression was unchanged, indicating different transcriptional regulation and perhaps function between the cathepsin D paralogues and the E3 Ub ligases. MAFbx mRNA levels decreased in gilthead sea bream muscle with ontogeny, as previously observed in rat gastrocnemius muscle during the age-related loss of muscle mass [74]. On the other hand, lack of transcriptional regulation of MuRF1 has been previously reported also in the human muscle of the elderly [75], in agreement with our results. Furthermore, SaUb mRNA expression was higher in fingerling and adult fish than juveniles. In mammals, a poor capacity to regenerate muscle with advanced age is thought to be due to impaired signalling, exhaustion of the satellite cell pool and/or changes in the extracellular matrix [26]. Consistent with this observation, a previous study found that Ub protein expression was also up-regulated in rat and human fast muscle fibres during aging, and it was shown using C2C12 muscle cells that Ub suppresses proliferation, which may be associated with the poor healing potential in older individuals [27]. The increase in SaUb expression between juveniles and adults may reflect the early stages of a similar age-related impairment of regenerative capacities in gilthead sea bream. However, Ub has been shown to have numerous proteolytic functions, including the regulation of proteasomal and lysosomal degradation, and also non-proteolytic functions, such as the regulation of protein interactions, activity and localization within the cell [76]. Thus, the significance of increased SaUb expression in adult fish may well be related to other factors and needs to be further explored.

qPCR analyses revealed a general significant increase and decrease in the expression of all the studied SaCTSs and SaUbP genes with fasting and re-feeding, respectively, with the only exception of SaCTSB that was unaffected in response to fasting. In a recent study on gilthead sea bream using a similar experimental design, fasting had little effect on several catalytic and regulatory members of the calpain system whereas these genes were down-regulated with re-feeding [44]. Thus there are likely generalised decreases in proteolytic enzyme expression in this species under anabolic conditions, assuming changes in transcript abundance are reflected in protein levels. In agreement with the present data, in a previous study in rainbow trout, cathepsin B, D and S and genes from the UbP pathway were up-regulated during fasting, and were also significantly down-regulated following re-feeding [33], and the same was observed with regards to Atlantic salmon (*Salmo salar*) cathepsin L [77], and the E3 ligases, MAFbx and MuRF1 in zebrafish [21], Atlantic salmon [22,77-79], pacu (*Piaractus mesopotamicus*) [80] and gilthead sea bream [2]. Similarly in another study in rainbow trout, the mRNA expression of the Ub-ligase MAFbx and the level of poly-ubiquitinated proteins in the muscle was significantly increased with fasting and decreased after re-feeding, although major changes were not observed in the activity of the main proteasomal peptidases (trypsin and chymotrypsin-like) [34]. Moreover in the same species, another study has demonstrated that the expression of MuRF genes was up-regulated with fasting as well as during spawning, suggesting an important role of the UbP system in fish during situations of induced muscle atrophy [81].

## Conclusions

In summary, the present study has shown that the different proteolytic systems are transcriptionally regulated during ontogeny and according to the physiological status in gilthead sea bream muscle, and adds to the evidence that feeding regimes and/or diet can be used to alter proteolysis in the muscle of farmed fish. This may provide a practical means of manipulating the extent of protein breakdown during *post-mortem* storage of fish so as to reduce the problems of soft flesh and gaping, which reduce economic value.

## Additional file

Additional file 1: Figure S1. Nucleotide and deduced amino acid sequences of gilthead sea bream cathepsin B (SaCTSB, [GenBank: KJ524457]). **Figure S2.** Nucleotide and deduced amino acid sequences of a new paralogue of gilthead sea bream cathepsin D (SaCTSDb, [GenBank: KJ524456]). **Figure S3.** Partial nucleotide and deduced amino acid sequences of gilthead sea bream proteasome subunit beta type-4 (SaN3, also known as PSMB4, [GenBank: KJ524458]). **Figure S4.** Nucleotide and deduced amino acid sequences of gilthead sea bream ubiquitin (SaUb, [GenBank: KJ524459]).
