# Supplementary material for: Characterisation and expression analysis of cathepsins and ubiquitin-proteasome genes in gilthead sea bream (Sparus aurata) skeletal muscle
Source: BMC Res Notes. 2015 Apr 15;8:149. doi: 10.1186/s13104-015-1121-0 (PMC4431372; doi:10.1186/s13104-015-1121-0)
Supplement: Additional file 1: Figure S1. — Nucleotide and deduced amino acid sequences of gilthead sea bream cathepsin B (SaCTSB, [GenBank: KJ524457]). Figure S2. Nucleotide and deduced amino acid sequences of a new paralogue of gilthead sea bream cathepsin D (SaCTSDb, [GenBank: KJ524456]). Figure S3. Partial nucleotide and deduced amino acid sequences of gilthead sea bream proteasome subunit beta type-4 (SaN3, also known as PSMB4, [GenBank: KJ524458]). Figure S4. Nucleotide and deduced amino acid sequences of gilthead sea bream ubiquitin (SaUb, [GenBank: KJ524459]). [file 13104_2015_1121_MOESM1_ESM.pdf]

Sup. Figure 1

```
  1 ATGTGGCGTGCAGCCTTCCTGTTATTGGCTGCCAGCTTGTCTGTGAGCCTGGCCAGACCC
  1  M  W  R  A  A  F  L  L  L  A  A  S  L  S  V  S  L  A  R  P

 61 CACCTCAAACCACTGTCCAATGAGATGGTCAACTACATCAATAAGTTTAACACTACCTGG
 21  H  L  K  P  L  S  N  E  M  V  N  Y  I  N  K  F  N  T  T  W

121 AAGGCTGGTCACAACTTTCATAATGTCGACTACAGTTATGTCCAGAGACTCTGCGGTACG
 41  K  A  G  H  N  F  H  N  V  D  Y  S  Y  V  Q  R  L  C  G  T

181 ATGCTGAAGGGACCTAAACTGCCCGTTATGGTTCAGTATGCTGGTGACCTGGAGCTTCCA
 61  M  L  K  G  P  K  L  P  V  M  V  Q  Y  A  G  D  L  E  L  P

241 AAAGAGTTTGACTCCAGAGTGCAGTGGCCCAACTGTCCCACTCTGAAGGAGATCAGAGAC
 81  K  E  F  D  S  R  V  Q  W  P  N  C  P  T  L  K  E  I  R  D

301 CAGGGCTCCTGTGGATCCTGCTGGGCGTTTGGTGCTGCAGAGGCCATCTCCGACCGTGTG
101  Q  G  S  C  G  S  C  W  A  F  G  A  A  E  A  I  S  D  R  V

361 TGTATCCACAGCAATGCCAAGGTCAGCGTGGAGATCTCCTCCGAGGATCTGTTGACATGC
121  C  I  H  S  N  A  K  V  S  V  E  I  S  S  E  D  L  L  T  C

421 TGTGACAGCTGTGGCATGGGATGTAATGGTGGCTACCCTTCAGCTGCCTGGGACTTCTGG
141  C  D  S  C  G  M  G  C  N  G  G  Y  P  S  A  A  W  D  F  W

481 ACCAAAGACGGGCTGGTCTCTGGAGGCCTCTATGATTCCCATGTCGGTTGTCGTCCCTAC
161  T  K  D  G  L  V  S  G  G  L  Y  D  S  H  V  G  C  R  P  Y

541 ACCATCGCCCCCTGCGAGCACCATGTGAATGGCAGTAGACCCCCTTGCACCGGAGAAGGT
181  T  I  A  P  C  E  H  H  V  N  G  S  R  P  P  C  T  G  E  G

601 GGAGAAACACCCCAGTGCATCTTCCAGTGTGAAGCTGGATACACACCAAGCTACAAACAA
201  G  E  T  P  Q  C  I  F  Q  C  E  A  G  Y  T  P  S  Y  K  Q

661 GACAAGCACTATGGTAAAACGTCTTACAGCGTGCTGTCGGATGAGGAGCAGATTCAGTAC
221  D  K  H  Y  G  K  T  S  Y  S  V  L  S  D  E  E  Q  I  Q  Y

721 GAGATATACAAGAATGGCCCAGTAGAGGGAGCCTTTATAGTCTATGAAGACTTTGTGCTG
241  E  I  Y  K  N  G  P  V  E  G  A  F  I  V  Y  E  D  F  V  L

781 TACAAGTCTGGTGTGTATCAGCATGTGTCTGGCTCTCAAGTTGGCGGCCATGCCATTAAG
261  Y  K  S  G  V  Y  Q  H  V  S  G  S  Q  V  G  G  H  A  I  K

841 ATCCTGGGCTGGGGGGAGGAGGCCGGTGTTCCCTACTGGCTCTGTGCCAACTCCTGGAAC
281  I  L  G  W  G  E  E  A  G  V  P  Y  W  L  C  A  N  S  W  N

901 ACGGACTGGGGTGATAACGGATTCTTTAAGTTCCTGCGTGGATCTGATCACTGTGGTATT
301  T  D  W  G  D  N  G  F  F  K  F  L  R  G  S  D  H  C  G  I

961 GAGTCTGAAATTGTGGCAGGAATTCCCAAGTAA
321  E  S  E  I  V  A  G  I  P  K  *
```

Sup. Figure 2

```
   1 ATGAGGAGCCTGGTCCTGTTCGTGTTCGCGGCGCTGGTTCTGACCACCGACGCGCTGGTT
   1  M  R  S  L  V  L  F  V  F  A  A  L  V  L  T  T  D  A  L  V

  61 CGAATTCCCTTAAAGAAATTCCGTTCCATCAGACGTGAGCTGACGGACTCGGGGAGGACC
  21  R  I  P  L  K  K  F  R  S  I  R  R  E  L  T  D  S  G  R  T

 121 GCAGAGGAGCTCCTGGCTGGCAAACACTCCACTAAGTACAACTTTGGCTTCCCCTCCAGC
  41  A  E  E  L  L  A  G  K  H  S  T  K  Y  N  F  G  F  P  S  S

 181 AATGCACCCACTCCAGAAACCCTGAAGAACTACCTTGACGCGCAGTATTACGGCGAGATC
  61  N  A  P  T  P  E  T  L  K  N  Y  L  D  A  Q  Y  Y  G  E  I

 241 GGCCTGGGGACTCCTCCTCAGCTCTTCACTGTGGTGTTTGATACGGGCTCCTCCAACCTG
  81  G  L  G  T  P  P  Q  L  F  T  V  V  F  D  T  G  S  S  N  L

 301 TGGGTGCCCTCCGTTCACTGCTCCCTCTTAGACATCGCCTGCTTGCTTCACCACAAATAT
 101  W  V  P  S  V  H  C  S  L  L  D  I  A  C  L  L  H  H  K  Y

 361 AATTCTGCCAAGTCCAGCACATACGTGAAGAACGGCACCGCCTTTGCAATCCAGTATGGA
 121  N  S  A  K  S  S  T  Y  V  K  N  G  T  A  F  A  I  Q  Y  G

 421 TCTGGCAGTCTGTCGGGCTACCTCAGTCAGGACACATGCACAATCGGAGACATCGCGGTG
 141  S  G  S  L  S  G  Y  L  S  Q  D  T  C  T  I  G  D  I  A  V

 481 GAAAAACAGCTTTTCGGAGAAGCCATCAAGCAGCCCGGTGTGACCTTCATCGCTGCCAAG
 161  E  K  Q  L  F  G  E  A  I  K  Q  P  G  V  T  F  I  A  A  K

 541 TTTGACGGCATCCTCGGCATGGCCTACCCACGCATCTCTGTGGACGGTGTGGCTCCCGTC
 181  F  D  G  I  L  G  M  A  Y  P  R  I  S  V  D  G  V  A  P  V

 601 TTTGACAACATCATGAGCCAGAAGAAGGTGGAGAAGAACGTCTTCTCCTTCTACCTGAAC
 201  F  D  N  I  M  S  Q  K  K  V  E  K  N  V  F  S  F  Y  L  N

 661 AGGAACCCCGACACCGAGCCCGGCGGTGAGCTGCTGCTCGGAGGGACTGACCCCAAATAC
 221  R  N  P  D  T  E  P  G  G  E  L  L  L  G  G  T  D  P  K  Y

 721 TACAGCGGAGACTTCAACTACGTCAACATCACCCGCCAGGCGTACTGGCAGATCCACATG
 241  Y  S  G  D  F  N  Y  V  N  I  T  R  Q  A  Y  W  Q  I  H  M

 781 GACGGGATGTCAGTGGGAACCCAGCTGAGTCTGTGTGGGAGCGGCTGTGAAGCCATCGTG
 261  D  G  M  S  V  G  T  Q  L  S  L  C  G  S  G  C  E  A  I  V

 841 GACACCGGGACGTCTCTGATCACCGGACCCTCAGCGGAGGTCAGGTCCCTGCAGAAAGCC
 281  D  T  G  T  S  L  I  T  G  P  S  A  E  V  R  S  L  Q  K  A

 901 ATCGGAGCCACTCCACTCATCCAGGGAGAGTACATGGTGAGCTGTGACAAAGTCCCGACG
 301  I  G  A  T  P  L  I  Q  G  E  Y  M  V  S  C  D  K  V  P  T

 961 CTGCCTGTCATCACCTTCAAAGTTGGCGGACAGTCTTACTCTCTGACCGGAGAGCAGTAC
 321  L  P  V  I  T  F  K  V  G  G  Q  S  Y  S  L  T  G  E  Q  Y

1021 ATCCTCAAGGTGAGTCAGGCTGGAAAGACCATGTGTCTGAGCGGCTTCATGGGTCTGGAC
 341  I  L  K  V  S  Q  A  G  K  T  M  C  L  S  G  F  M  G  L  D

1081 ATCCCCGCCCCCGCCGGGCCCCTGTGGATTCTGGGAGACGTCTTCATCGGCCAGTACTAC
 361  I  P  A  P  A  G  P  L  W  I  L  G  D  V  F  I  G  Q  Y  Y

1141 ACCGTCTTCGATCGGGACAACAACCGGGTCGGCTTCGCCAAGTCTAAATAA
 381  T  V  F  D  R  D  N  N  R  V  G  F  A  K  S  K  *
```

Sup. Figure 3

```
  3 GGTTTGAAGCTGAGTTTCTGGGAGAACGGACCGAAGCCCGGCCAGTTCTACTCTTTCCCC
  1  G  L  K  L  S  F  W  E  N  G  P  K  P  G  Q  F  Y  S  F  P

 63 GGCGGCAGCAGCAGCAGCGGGTCGGCAGCATCATGCGGGCCGGTCAGACACACACTGAAC
 21  G  G  S  S  S  S  G  S  A  A  S  C  G  P  V  R  H  T  L  N

123 CCGATGGTCACAGGGACGTCGGTGCTCGGTGTGAAGTTCACCGGCGGCGTCATCATCGCG
 41  P  M  V  T  G  T  S  V  L  G  V  K  F  T  G  G  V  I  I  A

183 GCGGACATGTTGGGCTCGTACGGCTCTCTGGCTCGCTTCAGGAACATCTCTCGTCTCATG
 61  A  D  M  L  G  S  Y  G  S  L  A  R  F  R  N  I  S  R  L  M

243 AAGGTGAACGGTAACACCATCCTGGGAGCGTCCGGAGACTACGCCGACTACCAGTACCTC
 81  K  V  N  G  N  T  I  L  G  A  S  G  D  Y  A  D  Y  Q  Y  L

303 AAACAGATCATCGAACAGATGGTTATCGACGAGGAGCTGCTGGGTGACGGTCACAGCTAC
101  K  Q  I  I  E  Q  M  V  I  D  E  E  L  L  G  D  G  H  S  Y

363 AGTCCCAAGGCGGTCCACTCCTGGCTCACCAGAGTCATGTACAACCGGCGCTGCAAGATG
121  S  P  K  A  V  H  S  W  L  T  R  V  M  Y  N  R  R  C  K  M

423 AATCCTCTGTGGAACACGGTGGTGATCGGAGGCTTCTACAACGGAGAGAGTTTCCTAGGT
141  N  P  L  W  N  T  V  V  I  G  G  F  Y  N  G  E  S  F  L  G

483 TACGTGGACAAGCTGGGCGTGGCCTATGAGGCGCCCACAGTGGCCACAGGCTTTGGAGCG
161  Y  V  D  K  L  G  V  A  Y  E  A  P  T  V  A  T  G  F  G  A

543 TACCTGGCTCAGCCTCTGATGAGGGAGGTGGTGGAGAACAAGGTGGAGATCACTAAGCAG
181  Y  L  A  Q  P  L  M  R  E  V  V  E  N  K  V  E  I  T  K  Q

603 GAGGCTCGGGAGCTAATCGAGCGCTGCCTCAAAGTGCTTTACTACAGAGACGCTCGCTCC
201  E  A  R  E  L  I  E  R  C  L  K  V  L  Y  Y  R  D  A  R  S

663 TACAACAGATACGAGATCGCCATCGTCACAGAGGAGGGCGTGGAGATCGTCGGTCCGCTG
221  Y  N  R  Y  E  I  A  I  V  T  E  E  G  V  E  I  V  G  P  L

723 TCTTCTGAGACCAACTGGGACATCGCTCACATGTCA
241  S  S  E  T  N  W  D  I  A  H  M  S
```

Sup. Figure 4

```
1   ATGCAGATCTTTGTAAAGACCCTGACTGGGAAGACCATCACTTTGGAGGTGGAGCCAAGT
1    M  Q  I  F  V  K  T  L  T  G  K  T  I  T  L  E  V  E  P  S

61  GACACCATTGAAAATGTGAAGGCTAAGATCCAGGATAAGGAGGGCATCCCCCCAGACCAG
21   D  T  I  E  N  V  K  A  K  I  Q  D  K  E  G  I  P  P  D  Q

121 CAGCGTCTGATCTTCGCTGGAAAGCAGCTTGAAGATGGTCGCACCCTCTCCGACTACAAC
41   Q  R  L  I  F  A  G  K  Q  L  E  D  G  R  T  L  S  D  Y  N

181 ATCCAGAAGGAGTCCACCCTCCATCTTGTGCTGCGTCTGAGGGGAGGAATGCAGATCTTC
61   I  Q  K  E  S  T  L  H  L  V  L  R  L  R  G  G  M  Q  I  F

241 GTGAAGACCCTGACTGGCAAGACCATCACCTTGGAGGTGGAGCCAAGTGACACCATCGAG
81   V  K  T  L  T  G  K  T  I  T  L  E  V  E  P  S  D  T  I  E

301 AATGTGAAGGCTAAGATCCAGGATAAGGAGGGCATCCCCCCAGACCAGCAGCGTCTGATC
101  N  V  K  A  K  I  Q  D  K  E  G  I  P  P  D  Q  Q  R  L  I

361 TTCGCTGGCAAGCAGCTTGAAGATGGTCGCACCCTCTCCGACTACAATATCCAGAAGGAA
121  F  A  G  K  Q  L  E  D  G  R  T  L  S  D  Y  N  I  Q  K  E

421 TCCACTCTTCACCTGGTCTTGCGTCTGAGGGGAGGAATGCAGATCTTTGTGAAAACCCTG
141  S  T  L  H  L  V  L  R  L  R  G  G  M  Q  I  F  V  K  T  L

481 ACTGGCAAGACCATCACCCTGGAGGTGGAGCCAAGTGACACCATCGAGAACGTGAAGGCT
161  T  G  K  T  I  T  L  E  V  E  P  S  D  T  I  E  N  V  K  A

541 AAGATCCAGGATAAGGAGGGCATCCCCCCAGACCAGCAGCGTCTGATCTTCGCTGGCAAG
181  K  I  Q  D  K  E  G  I  P  P  D  Q  Q  R  L  I  F  A  G  K

601 CAGCTTGAAGATGGCCGCACCCTCTCCGACTACAACATCCAGAAGGAGTCCACCCTCCAT
201  Q  L  E  D  G  R  T  L  S  D  Y  N  I  Q  K  E  S  T  L  H

661 CTTGTGCTGCGCCTCAGGGGAGGAATGCAGATCTTCGTGAAGACCTTGACTGGCAAGACC
221  L  V  L  R  L  R  G  G  M  Q  I  F  V  K  T  L  T  G  K  T

721 ATTACCTTGGAGGTGGAGCCAAGTGACACCATCGAGAACGTGAAGGCTAAGATCCAGGAT
241  I  T  L  E  V  E  P  S  D  T  I  E  N  V  K  A  K  I  Q  D

781 AAGGAGGGCATCCCCCCAGACCAGCAGCGTCTGATCTTCGCTGGCAAGCAGCTTGAAGAT
261  K  E  G  I  P  P  D  Q  Q  R  L  I  F  A  G  K  Q  L  E  D

841 GGTCGCACCCTTTCCGACTACAACATCCAGAAGGAATCCACCCTCCATCTTGTGCTCCGT
281  G  R  T  L  S  D  Y  N  I  Q  K  E  S  T  L  H  L  V  L  R

901 CTGAGGGGAGGAAACTAA
301  L  R  G  G  N  *
```
